# Adult diffuse hepatic hemangiomatosis lesion occupying the entire abdominal and pelvic cavities: a case report

**DOI:** 10.3389/fmed.2024.1399913

**Published:** 2024-09-19

**Authors:** Ya-Nan Ge, Yan Shao, Shu-Chen Dong, Xing-Bin Ma, Wei Wang

**Affiliations:** ^1^Binzhou Medical University Hospital, Binzhou, China; ^2^Shandong University of Aeronautics, Binzhou, Shandong, China

**Keywords:** diffuse hepatic hemangiomatosis, giant cavernous hemangioma, imaging features, Kasabach-Merritt syndrome, multimodality evaluation

## Abstract

**Introduction:**

Adult diffuse hepatic hemangiomatosis (DHH) is an extremely rare disease. Consequently, its characteristics are poorly understood. Herein, we report a case of adult DHH involving both liver lobes but without extrahepatic involvement. To the best of our knowledge, this the largest reported adult DHH to date.

**Case presentation:**

A 51-year-old man was admitted due to abdominal distension and dyspnea. Physical examination revealed marked liver enlargement. Color Doppler, plain and contrast-enhanced computed tomography, and contrast-enhanced magnetic resonance imaging revealed a hepatic lesion sized 35.1 × 32.1 × 14.1 cm occupying nearly the entire abdominal and pelvic cavities. Diagnosis was established by liver puncture biopsy. The patient exhibited clinical signs of portal hypertension and hypersplenism, but remains free of serious DHH-related complications. He is followed up regularly, with proactive evaluation for future liver transplantation.

**Conclusion:**

This case will contribute to the current knowledge on the clinical and imaging features of this rare entity.

## 1 Introduction

Hepatic hemangiomas are the most common benign tumors of the liver, with an incidence of 5–20% (1). In most cases, they are isolated, asymptomatic, and incidentally detected. Contrarily, diffuse hepatic hemangiomatosis (DHH) is a rare disease that usually develops in newborns and is characterized by extensive replacement of the liver parenchyma with hemangiomatous lesions (2). The difference between DHH and multiple or giant hepatic hemangiomas is that, in the former, the lesions have poorly defined margins with diffuse replacement of hepatic tissue, whereas in the latter, hemangiomas are surrounded by a fibrous capsule with well-defined margins. Adult DHH, particularly without extrahepatic involvement, is extremely rare.

Herein, we present a case of adult DHH without extrahepatic involvement presenting with extremely enlarged liver occupying the entire abdominal and pelvic cavities, and describe its imaging features as determined by multimodality evaluation.

## 2 Case presentation

A 51-year-old man was hospitalized on May 31, 2023 due to abdominal distension lasting for 3 years and chest tightness lasting for 1 week. Three years previously, the patient was diagnosed with venous thrombosis of the right lower extremity and had been taking rivaroxaban for an extended period. He had no history of hepatitis, denied a history of alcoholism, and had diabetes and hypertension. He did not have telangiectasia of the lips or mouth and denied episodes of epistaxis.

Physical examination revealed markedly enlarged liver occupying nearly the entire abdominal and pelvic cavities, with the lower margin reaching the superior margin of the pubic symphysis.

Complete blood count analysis revealed leukopenia and thrombocytopenia, with a white blood cell count of 2.9 × 109/L (reference range: 4–10 × 109/L) and platelet count of 80 × 109/L (reference range: 100–300 × 109/L). Liver function and coagulation function test results were normal. Tests for hepatitis A, B, C, D, and E, Epstein–Barr virus, cytomegalovirus, liver immunological markers, and ceruloplasmin yielded negative results. Parasitic infection was ruled out based on epidemiological history. Serum tumor marker levels (cancer antigen 125, alpha fetoprotein, carcinoembryonic antigen, cancer antigen 199, total prostate-specific antigen, and squamous cell carcinoma antigen) were within normal ranges.

**Figure 1:**
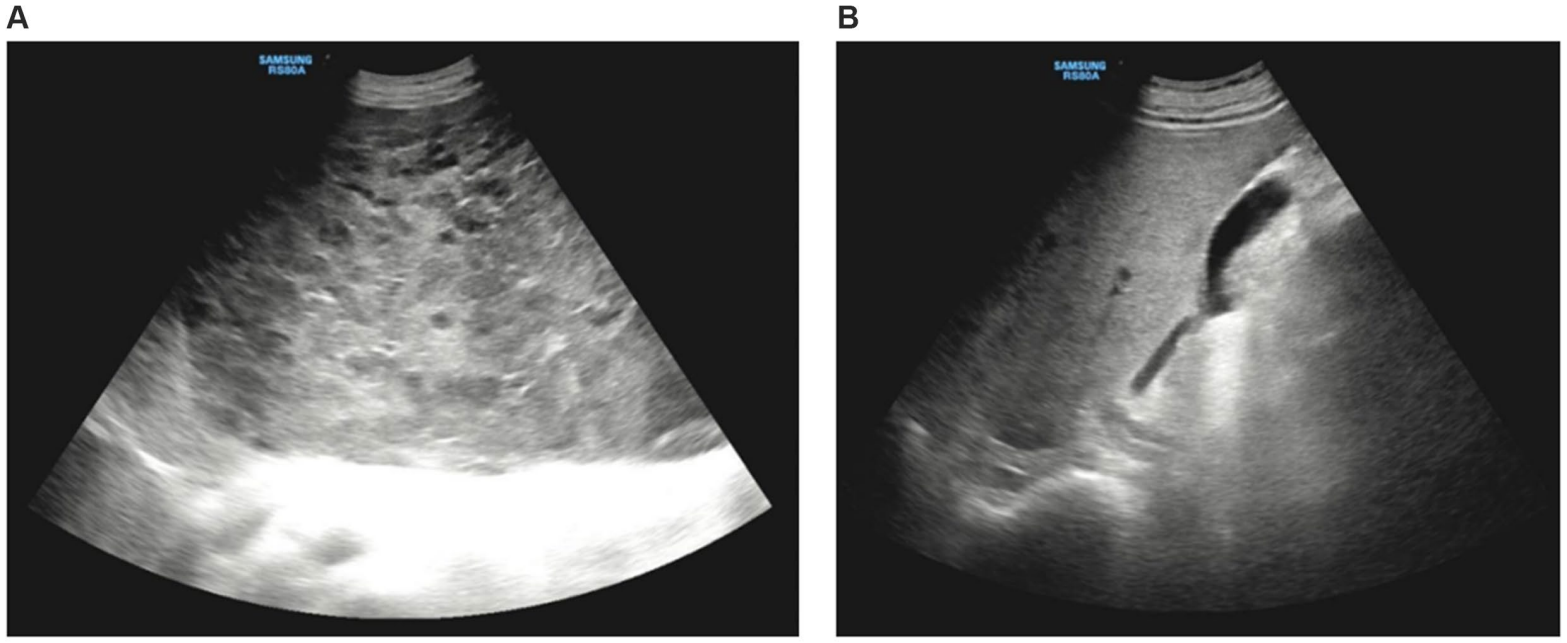
Doppler ultrasound imaging. **(A)** Ultrasound showed huge occupation in the liver, with uneven internal echoes, and morphological under-rule. **(B)** The normal liver tissue in the right lobe of liver.

Abdominal Doppler ultrasound examination suggested massive liver enlargement. The liver capsule was smooth, and a giant space-occupying lesion was detected. The lesion had irregular morphology and protruded from the liver, extending to the xiphoid process superiorly, 8.0 cm below the umbilicus inferiorly, and the posterior axillary line on the left side. The liver showed inhomogeneous echogenicity, with only a small amount of normal liver tissue remaining in the right lobe (Figure 1).

**Figure 2:**
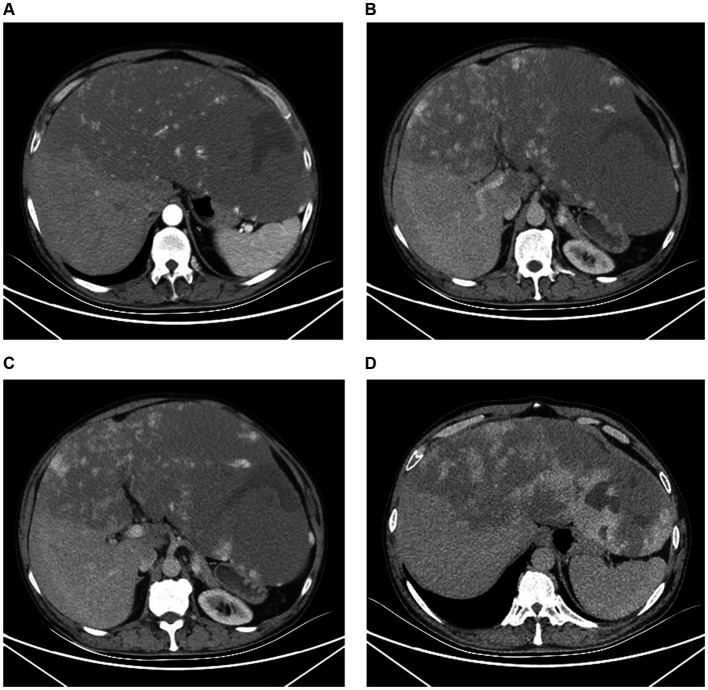
Computed tomography imaging. **(A)** The arterial phase showed the irregular patchy foci of enhancement. **(B,C)** The portal venous phase expressed the range of enhancing gradually increases. **(B)** The left portal vein branch was extremely thin and the right portal vein branch become thinner. **(C)** The main portal vein in hepatic hilar region was slightly narrowed. **(D)** The delayed phases showed uniform enhancement of most of the lesion and patchy low-density shadows in the lateral side of the left lobe.

Contrast-enhanced abdominal computed tomography (CT) revealed a massive area of abnormal enhancement in the liver parenchyma partially protruding from the contour of the liver and growing toward the abdominal cavity. The lesion covered an area of approximately 32.4 × 32.2 × 16.2 cm, with only a small amount of normal liver tissue remaining in the right lobe. In the arterial phase, the lesion contained multiple patchy foci of significant enhancement. In the portal venous and delayed phases, the extent of enhancement increased with increased scanning time. Delayed scanning after about 30 min showed uniform enhancement of most of the lesion and patchy low-density shadows in the lateral side of the lesion. The portal vein was slightly narrowed by compression, there was no significant dilation of the splenic vein, and no significant splenomegaly (Figure 2).

**Figure 3:**
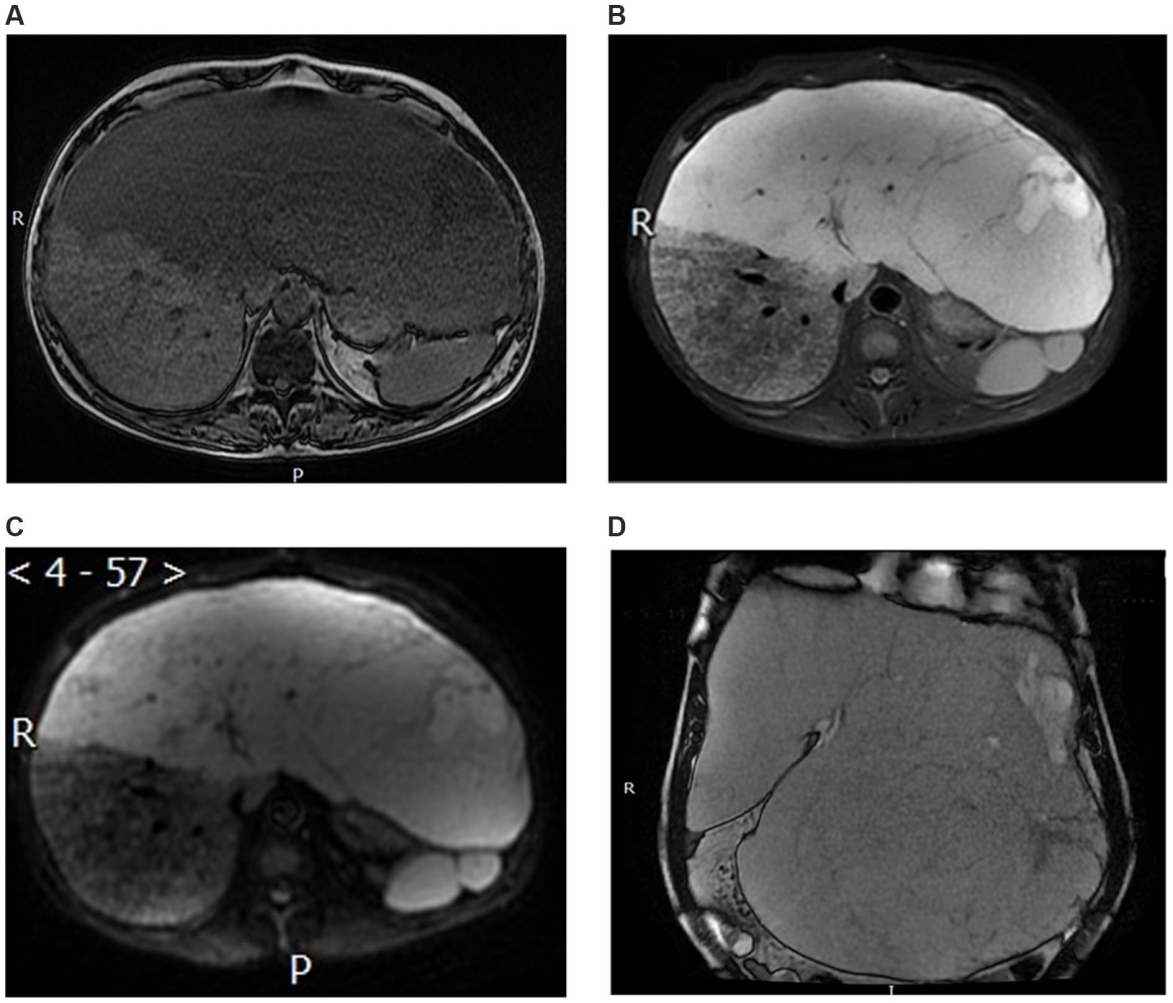
Magnetic resonance imaging. **(A)** T1-weighted image. **(B)** T2-weighted image. **(C)** Apparent diffusion coefficient image. **(D)** The coronal MRI revealed enlarged liver occupying nearly the entire abdominal and pelvic cavities.

Contrast-enhanced abdominal magnetic resonance imaging (MRI) showed marked liver enlargement with a mass-like abnormal signal in the left lobe and most of the right lobe. The lesion was slightly hypointense on T1-weighted images, slightly hyperintense on T2-weighted and diffuse-weighed images, and locally hypointense on the apparent diffusion coefficient map. The left lobe exhibited local streaks with even lower T1 and even longer T2 signals, with streaks of hypointense segments within. The lesion had clear margins, measured 35.1 × 32.1 × 14.1 cm, and partially protruded from the liver contour, compressing the surrounding structures (Figure 3).

**Figure 4:**
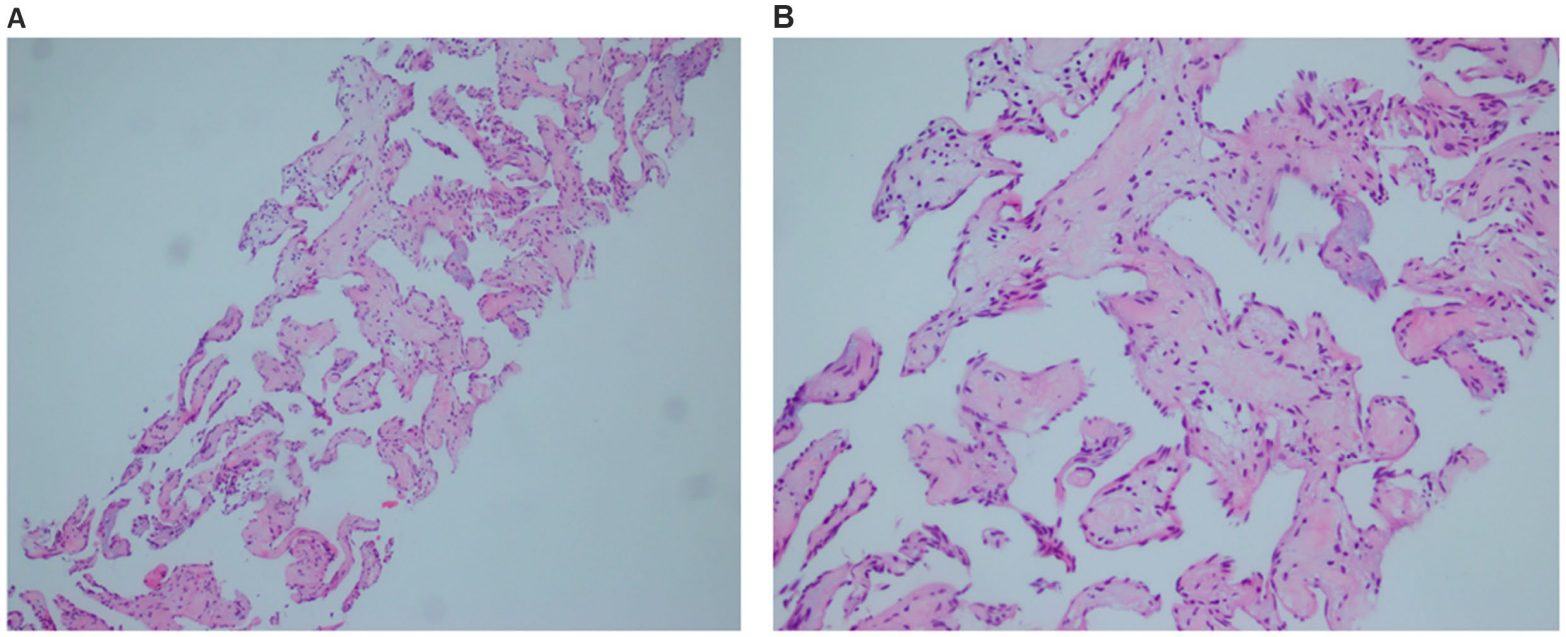
Pathological image. Histological section showing that the lesion consisted of cystic dilated vessels of variable size, with large and irregular lumens and uneven wall thickness, and were lined with flattened endothelial cells with no cellular heterogeneity. **(A)** The low magnification. **(B)** The higher magnification.

To confirm the diagnosis, ultrasound-guided biopsy of the right liver lobe was performed. Histopathological analysis revealed that the lesion consisted of cystic dilated vessels of variable size, with large and irregular lumens and uneven wall thickness, and were lined with flattened endothelial cells with no cellular heterogeneity (Figure 4).

Our differential diagnoses included diffuse hamartoma, multiple metastases, polycystic iver, diffuse hepatocellular carcinoma, angiosarcoma, and hemangioendothelioma.

Based on the histopathological and imaging findings, the patient was diagnosed with adult DHH. At present, no effective treatment is available for DHH with near-total liver involvement. The patient is free of significant complications, such as liver failure, high-output heart failure, Kasabach-Merritt syndrome, or disseminated intravascular coagulation. Therefore, regular follow-up is performed with consideration for future liver transplantation. At nearly 6 months of follow-up, the patient remains stable with intermittent epigastric pain.

## 3 Discussion

Adult DHH is a rare disease, with only 20 reported cases as of August 2023 (Table 1), over half of which were associated with giant cavernous hemangioma (GCH) (3). In the present case, DHH was not accompanied by GCH, but was diffusely distributed throughout the liver, manifesting as poorly defined spongy vascular tissue occupying nearly the entire liver. The involvement of the left lobe was particularly pronounced. It was completely occupied by hemangiomatous tissue, resulting in a marked increase in its size, reaching the superior margin of the pubic symphysis inferiorly and the posterior axillary line on the left side. The enlarged liver occupied nearly the entire abdominal and pelvic cavities, reaching a size of 35.1 × 32.1 × 14.1 cm. To the best of our knowledge, this is the largest DHH reported to date.

**Table 1:** The largest diffuse hepatic hemangiomatosis lesion in recent literature.

Ref	Age/Sex	Clinical features	GCH	Reaching	Histopathology	Treatment	Outcome
(19)	56/F	Generalized bone pain, abdominal pain and abdominal swelling due to hepatic mass	NA	The liver ex-tended 10 cm below the costal margin	Liver and bone with numerous capillary angiomas consistent with hemangiomatosis. Cystic spaces lined by thin, homogeneously stained endothelial cells. Areas of tumor with numerous intraluminal red blood cells.	Prednisone	Died after 2 weeks
(20)	30/F	Hepatosplenomegaly, anemia and thrombocytopenia	NA	The liver was palpable two fingerbreadths below the navel and the spleen three fingerbread-ths below the left costal margin.	Hepatic parenchyma replaced by hemorrhagic cystic tumors. Similar vascular lesion was seen in the spleen, bone marrow, intestine, and peripancreatic lymph nodes.	Radiotherapy Splenectomy	Died 12 months after first presentation due to consumptive coagulopathy
(21)	22/F	Right upper abdominal pain	NA	Ultra-sonography disclosed an enlarged liver with numerous echogenic figures approximately 1.5 cm in diameter with homogenous internal ethos	Numerous irregularly formed slit-like blood vessels lined by endothelium and slight focal portal fibrosis close to the central vein. The portal triads showed capillary sprouting. The liver capsule was retracted by bands containing capillaries and collagenous fibers.	Stoppage of drug	Regression of lesions
(10)	35/F	Abdominal pain, weight loss, night sweats and fever	NA	CT demonstrated a large, ill-defined, hypoatten-uation mass with areas of hemorrhage in the left he-patic lobe, indicating the finding of a presumably ma-lignant tumor.	Cavernous and diffuse, hemangiomatosis and hemorrhage within the tumor. Innumerable, confluent vascular channels lined by flat endothelial cells linked the hemangiomas.	Left hepatectomy	Recurrence and growth into the right hepatic lobe after 2 years of surgery. Still progressing at 6 years of follow up.
(14)	50/F	Tenderness in right upper abdominal quadrant	Yes	The specimen measured 17× 14×9 cm in dimension and multiple hemorrhagic bloodfilledhoneycomb areas from 2 to 3 mm up to 3 cm in diameterwere scattered throughout entire right lobe.	Cavernous hemangioma surrounded by hepatic parenchyma. Vascular channels lined by flattened endothelial cells. No cellular atypia	Right hepatectomy	No mass detected on ultrasonography 9 months post-surgery
(22)	33/F	Abdominal distension, edema and hepatomegaly	NA	An ultrasonography revealed a diffuse heterogeneous echoic infiltrative mass involving the entire liver	Prominent cavernous vascular proliferation and fibrosis without angiosarcomatous components	None	Patient expired due to liver failure 10 days after admission
(23)	33/F	Abdominal distension and shortness of breath	NA	NA	Variably dilated non-anastomotic vascular spaces lined by flat endothelial cells (CD 34 +).	NA	Died of liver failure after 12 days while waiting for liver transplant
(24)	78/M	Abdominal pain and distension	Yes	The CT demonstrated diffuse hepatic nodules involving the entire liver and a large heterogeneous mass replacing the left medial segment and caudate lobe	Cavernous hemangioma with irregularly dilated non-anastamotic vascular spaces lined by flat endothelial cells alternating with normal hepatic parenchyma	None	Improvement of discomfort and quality of life after 9 months follow up.
(15)	35/F	Epigastric pain and abdominal fullness	Yes	CT scan revealed a massive liver tumor in the right lobe of her liver, 153 mm × 133 mm × 95 mm in size.	Hemangiomatous lesions were scattered around the Glisson’s capsule	Right hepatectomy	Discharged on POD9 without identifiable lesions
(25)	68/M	Asymptomatic	NA	Ultrasound examination demonstrates multiple ill-defined yperechoic nodules dispersed throughout the hepatic parenchyma, mostly sub-centimeter and with right hemi-liver predominance.	Endothelial-lined sinusoidal proliferation with erythrocyte content, consistent with hepatic hemangiomatosis	None	Stable at 2 years of follow up
(11)	59/M	Asymptomatic	NA	A laparoscopic-guided biopsy was performed showing the liver occupying two thirds of his abdomen	H&E showed hemangiomas with (CD34+, CD31+, anti-desmin negative) no cellular atypia.	NA	Died due to hepatic failure
(17)	50/F	Abdominal pain, hepatomegaly and dyspnea	Yes	An abdomen CT demonstrated a 16 cm sized giant hemangioma in the central portion of the liver.	Main mass with variable-sized vascular spaces, lined by endothelial cells. Multiple scattered small hemangiomas also present.	Liver transplant	Stable for 1.5 years after surgery
(26)	83/F	Abdominal distension and hepatomegaly	NA	Contrast-enhanced dynamic abdomi- nal CT demonstrated diffuse, hypodense hepatic nodules with delayed enhancement involving the whole liver.	Irregularly dilated vascular spaces, mostly close to the portal tract, lined by endothelial cells (CD34+ and CD31+) without cellular atypia	Bevacizumab	Died 12 months after diagnosis due to multiple organ failure (KMS, hemolytic anemia, heart failure, DIC)
(27)	62/M	Asymptomatic	NA	A magnetic resonance imaging (MRI) of the liver showed multiple liver lesions of varying sizes in the right lobe, with the largest lesion measuring 1.8 × 1.6 cm at segment VII.	Focal areas of sinusoidal dilatations lined by flattened endothelial cells.	NA	NA
(9)	29/F	H/O Endometriosis and received OCPs	Yes	A 78-mm marginal hyperechoic mass in liver segment IVb and a 70-mm tumor in liver segment IVa.	Disseminated aggregate of blood vessels lined by endothelium without atypia.	Stoppage of drug and left liver lobectomy	No recurrent liver lesion after 1 year of surgery.
(18)	40/F	Abdominal pain and distension	Yes	When 2 CT scans done 8 months apart (March vs. November 2019) were compared, a 150% increase in the size of the segment 4 lesion was shown, to 32 × 23 × 20 cm, along with multiple small hemangiomas involving the entire liver	DHH with a giant hemangioma. No mitosis. STAT6, WT1, Desmin, p53, D2-40 were negative.	Chemotherapy, trans-arterial embolization and Liver transplant	Good condition after 6 months of follow-up.
(28)	63/M	Abdominal bloating and constipation	Yes	This hemangioma had a maximum transverse diameter of 21 cm and an anteroposterior diameter of 9 cm	NA	None	NA
(12)	62/M	Backache, Hepatomegaly	NA	A mass, approximately 47*39 mm in size a.	Lesion filled with red blood cells, lined by flat endothelial cells (CD34+) without atypia.	None	Good condition after 6 months of follow-up.
(29)	56/F	Chronic abdominal discomfort	Yes	a 19 × 18 × 16 cm well-defined mass	NA	NA	NA
(16)	37/F	Right upper quadrant fullness and shortness of breath	Yes	The contrast-enhanced computed tomography (CECT) scan showed an enlarged liver (liver span of 20 cm) with multiple heterogeneous hypodense lesions involving both the lobes.	Microscopic insinuation of the vascular lesion margins into the normal hepatic sinusoids. Lesions with variably-sized to large, thin-walled, non-anastomosing vascular spaces, and irregularly attenuated thick muscle walls. Vascular spaces lined by single layer of endothelial cells. Fibrin thrombi were noted within these vascular spaces. Stroma with fibrosis, myxoid degeneration, hyalinization, and calcification.	Surgical enucleation	Good condition after 6 months of follow-up.
Our case	51/M	abdominal distension and chest tightness	NA	The lesion covered an area of approximately 32.4 × 32.2 × 16.2 cm	Histopathological analysis revealed that the lesion consisted of cystic dilated vessels of variable size, with large and irregular lumens and uneven wall thickness, and were lined with flattened endothelial cells with no cellular heterogeneity	NA	Good condition after 6 months of follow-up.

DHH usually presents with abdominal pain and distension. Some patients may also develop dyspnea and jaundice. Dyspnea is primarily due to the space occupation of the abdominal cavity by the liver associated with restricted respiration due to diaphragmatic elevation, as in this case. Jaundice may be due to liver dysfunction caused by vascular disease, mass effect, and ischemia. Heart failure, liver failure, disseminated intravascular coagulation, and Kasabach-Merritt syndrome are serious complications of DHH (4) and the leading causes of patient death. Portal hypertension can develop in cases of diffuse DHH progression with portal vein compression (5). These tumors may cause a mass effect when they attain substantial dimensions or have a modified internal component, such as a thrombosis or hemorrhage, transforming the lesion into a firm solid mass (24). It may cause the intrahepatic portal vein obstruction. Mild portal vein compression was also observed in the present case. Although no significant splenomegaly was found, we considered that the leukopenia and thrombocytopenia that could not be explained by other disorders were due to portal hypertension-induced hypersplenism.

According to the imaging features, DHH can manifest as a multinodular type, consisting of multiple discrete or coalescing nodules, and diffuse type, consisting of numerous poorly demarcated lesions with a tendency to converge to the point of replacing the entire liver (6–8). Unlike in cases of multiple hepatic hemangiomas, the lesion margins in DHH are usually indistinct and not clearly demarcated from the normal liver tissue. On Doppler ultrasound, the lesions appear as homogeneously hyperechoic areas with poorly defined margins or multiple small hypoechoic nodules on a hyperechoic background, varying significantly between patients. The lesions lack typical peripheral enhancement on contrast-enhanced CT and tend to present as patchy or crescent-shaped enhancements. On dynamic contrast-enhanced MRI, the lesions are hypointense on T1-weighted and hyperintense on T2-weighted images, with restricted diffusion on diffusion-weighted images, and exhibit discontinuous centripetal fill-in on axial dynamic images. In the present case, the hemangiomatous tissue occupied nearly the entire liver, with a diffuse and continuous distribution, and CT and MRI results were consistent with the typical presentation described above. The lesion exhibited diffuse and slightly hypointense areas with homogeneous density on plain CT, which was not easily distinguished from normal liver tissue and differed from the more common diffuse DHH or GCH with DHH manifestations, leading to possible misdiagnosis without contrast-enhanced CT.

Due to the rarity of adult DHH, little is known about its etiology or natural history. DHH is among the hepatic manifestations of Rendu-Osler-Weber disease and skeletal hemangiomatosis (9). Prior studies have reported possible etiologic associations between DHH and medication use (e.g., metoclopramide, estrogens, and Chinese patent medicines) (9–12). The correlation between hypothyroidism and DHH has been described in infants but not in adults (13). Jhaveri et al. (3) reported that GCH can be accompanied by DHH. In the present case, there was no extrahepatic involvement, no history of medication use, including metoclopramide, estrogens, or Chinese patent medicines, and no GCH; thus, a clear etiology could not be established.

If the tumor borderline is clear and confined to one lobe, surgical resection can be performed. In the literature, five patients underwent surgery with right/left hepatectomy (9, 10, 14–16). Radiation and Anti-VEGF (anti-vascular endothelial growth fac-tor) treatment has also been reported. The liver transplantation is the last resort. Two patients underwent living-donor liver transplantation and was in good condition at the follow-up (17, 18).

## 4 Conclusion

The present case provides description of the clinical and imaging characteristic of adult DHH. We conducted multimodality evaluation, including abdominal color Doppler ultrasound, CT, MRI, and liver puncture biopsy and pathology. We believe that the described findings can help improve our understanding of this rare disease. Since the probability of serious complications is significantly higher in cases of near-total liver involvement and severe liver enlargement, continuous monitoring, regular reexamination, and proactive evaluation for liver transplantation are necessary.

## Data Availability

The original contributions presented in the study are included in the article/Supplementary material, further inquiries can be directed to the corresponding author.
